# Human Drug Pollution in the Aquatic System: The Biochemical Responses of *Danio rerio* Adults

**DOI:** 10.3390/biology10101064

**Published:** 2021-10-19

**Authors:** Manuela Mauro, Valentina Lazzara, Vincenzo Arizza, Claudio Luparello, Vincenzo Ferrantelli, Gaetano Cammilleri, Luigi Inguglia, Mirella Vazzana

**Affiliations:** 1 Dipartimento di Scienze e Tecnologie Biologiche, Chimiche e Farmaceutiche (STEBICEF), Università degli Studi di Palermo, 90128 Palermo, Italy; valentina.lazzara@community.unipa.it (V.L.); vincenzo.arizza@unipa.it (V.A.); claudio.luparello@unipa.it (C.L.); luigi.inguglia@unipa.it (L.I.); mirella.vazzana@unipa.it (M.V.); 2 Istituto Zooprofilattico Sperimentale della Sicilia A. Mirri, 90129 Palermo, Italy; gaetano.cammilleri86@gmail.com

**Keywords:** cortisol, immune response, oxidative stress, enzyme, pharmaceutically active compounds (PhAC), zebrafish

## Abstract

**Simple Summary:**

The release of medicinal products for human use in the aquatic environment is now a serious problem, and can be fatal for the organisms that live there. *Danio rerio* is a freshwater fish that provides the possibility to study the effects of these pollutants on the health of aquatic organisms. The results of the various existing scientific studies are scarce and conflicting. Here, we review the scientific studies that have analyzed these effects, highlighting that the impacts of drugs are evident in the biochemical responses of these animals.

**Abstract:**

To date, drug pollution in aquatic systems is an urgent issue, and *Danio rerio* is a model organism to study the toxicological effects of environmental pollutants. The scientific literature has analyzed the effect of human drug pollution on the biochemical responses in the tissues of *D. rerio* adults. However, the information is still scarce and conflicting, making it difficult to understand its real impact. The scientific studies are not consistent with each other and, until now, no one has grouped their results to create a baseline of knowledge of the possible impacts. In this review, the analysis of literature data highlights that the effects of drugs on adult zebrafishes depend on various factors, such as the tissue analyzed, the drug concentration and the sex of the individuals. Furthermore, the most influenced biochemical responses concern enzymes (e.g., antioxidants and hydrolase enzymes) and total protein and hormonal levels. Pinpointing the situation to date would improve the understanding of the chronic effects of human drug pollution, helping both to reduce it in the aquatic systems and then to draw up regulations to control this type of pollution.

## 1. Introduction

Several types of pollutants affect aquatic systems, such as polystyrene microspheres [1], thiacloprid [2], noise [3,4,5], heavy metals [6,7], organophosphate flame retardants [8], micro-plastic [9,10,11], pesticides [12,13] and pharmaceutical and personal care products [14]. These cause negative effects on organisms at different levels (physical, physiological and behavioural), compromising their immune responses and survival with a damaging impact on the aquatic biodiversity that is a valuable human resource of bioactive molecules with antibacterial activity [15,16] and is beneficial for treating human diseases [17,18,19,20,21,22,23]. In the literature, a number of scientific studies report different types of pollution affecting aquatic ecosystems, and, among these, the most studied is chemical pollution due to industrial discharges, agricultural drugs, cosmetics and pharmaceutical products against human diseases [24]. Such compounds have not been monitored adequately to date, and specific regulatory standards concerning disposal or maximum environmental levels do not exist [25]. A significant concern relates to pharmaceutically active compounds (PhACs) [26,27], whose consumption over the years has increased globally due to their widespread use in the treatment of diseases in zootechnics, aquaculture and human medicine [28,29,30]. Considerable amounts of drugs reach the aquatic systems through their inappropriate disposal via sinks, toilets and the discharges of hospitals and the pharmaceutical industry. Wastewater treatment plants (WWTPs) are expected to intervene to prevent increases in the levels of most drugs in aquatic environments, but their efforts to date have been inadequate when it comes to efficient and complete removal [31]. For all these reasons, PhACs are continuously released in surface waters and underground [32,33], and are omnipresent in WWTPs around the world [34,35,36]. Knowing the concentration of a drug in the aquatic environment is crucial, and literature studies report levels ranging from a few ng/L to mg/L in both wastewater and natural aquatic systems [37,38,39,40]. Among the drugs identified in aquatic systems, there are: (1) clonazepam, carbamezapine, diazepam, fluoxetine and venlafaxine, which are prescribed for epileptic, psychotic and anti-depressive diseases [41,42,43,44,45,46,47]; (2) gemfibrozil, used to treat hypercholesterolemia and hyperglycaemia disorders [26,48]; (3) diclofenac, ibuprofen and ketoprofen, which have anti-inflammatory and analgesic effects [49,50,51]; and (4) tetracyclines, amoxicillin and sulfamethoxazole, which have an antibiotic role [52,53,54,55,56]. As persistent contaminants, PhACs can be bioaccumulated and biomagnified in the food chain (even if many human medicines weakly accumulate in biota), meaning that drug pollution is a particularly urgent societal and environmental problem [57,58,59,60,61,62]. Indeed, the literature reports ecotoxicological effects on non-target animals, including tissue damage (e.g., in the gills, liver, muscle and plasma) and oxidative and metabolic stress [63,64,65]. Scientific studies have analysed the impact of acute exposures to high drug concentrations [63,64,66,67], with only a few evaluating the effects at sub-lethal concentrations (the report by Almeida et al. [68], which examined the effects of Oxytetracycline on *Danio rerio* adults, is an example of the latter). Furthermore, knowledge of the impact on the organs and tissues involved is still incomplete [66,68,69]. To date, some efforts have been made to regulate the presence of these pollutants in aquatic environments. For example, article 8c of the Priority Substances Directive (2008/105/EC as amended by Directive 2013/39/EU) requires the European Commission to propose a strategic approach to the pollution of water by these substances, and the commitment to this end has been maintained in 2019 with the Communication [70]. The approach supports the Commission’s aim of delivering a Europe that protects the environment [71] and that works towards a sustainable Europe by 2030, following the Sustainable Development Goals [72]. Moreover, in 2020, the Regulation (EU) 2020/741 of the European Parliament and of the Council was published [73], which establishes the minimum quality requirements to use reclaimed water both to protect the environment and human/animal health, and then to promote the circular economy. For example, while in some European countries (e.g., France) the presence and limits of pesticides in water is regulated by Council Directive 98/83/EC (European Council, 1998), to date, no regulation includes pharmaceutical residues. Although some emerging contaminants are among the research lines of the World Health Organization (WHO) and Environmental Protection Agency (EPA), no legal discharge limits are described in the current legislation, and only some of them (e.g., Diclofenac) are included in the European Union Watch List (Directive, 2013/39/EU, modified by Decision 2015/495/EU of 20 March 2015 and updated in Decision 2018/840/EU of 5 June 2018) (Directive 2013/39/EU; Jurado, et al., 2019). Moreover, only six families of molecules (17-alpha-ethinylestradiol, 17-betaestradiol, estrone, macrolide antibiotics, amoxicillin, ciprofloxacin) are included on the watch list of substances for Union-wide monitoring, established in 2018 by Implementing Decision (EU) 2018/840 (European Commission, 2018). Despite everything, the problem remains current and of interest to all the major countries, as evidenced by their legislation on drug pollution [74].

## 2. *Danio rerio* as a Model Organism

In recent decades, *Danio rerio* (Hamilton-Buchanan 1822), a cyprinid commonly known as the zebrafish, has exponentially been adopted among the scientific community because of its crucial genetic, anatomical and physiological homology with mammals, becoming a model organism for biomedical research due to its genetic similarity to human beings [74,75,76,77,78,79]. In fact, it shares a high level of genome structure with humans and, in detail, approximately 70% of human genes have at least one obvious zebrafish ortholog compared to 80% of human genes with mouse orthologs, thereby facilitating the use of zebrafish for understanding human genetic diseases [77,80]. This species is widely used to test the efficacy of some drugs [76,81,82,83,84] and as an indicator organism for the study of the toxicological effects of different types of environmental pollutants (i.e., pesticides, industrial discharges, cosmetics; [85,86,87,88,89,90,91,92,93]. It is also an important model being inexpensive, low-maintenance and producing abundant offspring [94]. For all these reasons, the zebrafish has key advantages over other aquatic species in relation to the study of the effects of aquatic pollutants [95], and standard toxicity tests have been developed and recommended by a variety of international organizations [96]. As reported by Cassar et al. [97], zebrafish are an ideal model for the identification of drugs’ toxicology in respect to the 3R values: replacement, reduction and refinement. This species also enables non-invasive studies of toxic mechanisms and, in these cases, its recovery is easily examinable. Moreover, the ease of generating transgenic individuals of *Danio rerio* allows the undertaking of toxicological studies, as well as gene expression and cell-specific reporter assays in real-time in vivo conditions. Scientific studies using zebrafish to examine the effects of drugs released into aquatic systems have analysed the impacts at the embryo and larval levels, highlighting, for example: delays in hatching and growth and the formation of hydroedema [66,98,99,100]; developmental and acute toxicity and impairment in individual behaviors [101,102,103]; and the loss of kinocilia in neuromasts [104].

Given the considerable number of studies in the literature dealing with the effects of drugs on zebrafish, in this review we have decided to analyse only the adult stage. This is because, as observed by Oliveira et al. [105], animals in such life stage may be more sensitive to drugs than embryos and enable chronic effects to be evaluated [106]. Moreover, compared to embryo or larval studies, one advantage of adult exposure is that it is possible to collect blood or tissue from individuals in order to observe tissue specific responses. Adult models are more convenient in linking biochemical level responses to adverse health outcomes at an organism level. The purpose of this review is to gather the scientific studies that have analysed the in vivo effects of human drugs released into the aquatic system on the tissues of adult zebrafishes (excluding juveniles and embryos) only at the biochemical level (enzymes, hormones, total protein and lipids), excluding the histopathological changes, the molecular responses and the effects on reproductive aspects (e.g., spawning, eggs production). The goal is to create a biochemical baseline of each drug’s effects at different concentrations and exposure times. In the literature, many works use the zebrafish model to study the potential of different drugs on humans [97] and to evaluate the toxicological effects of several type of pollutants on zebrafish [107], but no studies focused on the effects of human drugs released in the aquatic system on adult zebrafish at the biochemical level, which is the purpose of this review. Moreover, among the environmental biomarkers used to study and monitor pollution levels, biochemical responses (with ecologically meaning endpoints) are among the most important for assessing the risks and quality of an aquatic system [108], being that they provide information on the health of individual organisms and, therefore, ecosystems [109]. In the future, this could help with the design of experiments aimed to increase the knowledge of the effects of drugs, both generally and at particular concentrations, leading to the development and improvement of regulations for environmental monitoring.

## 3. Biochemical Effects on Zebrafish

### 3.1. Antibiotics and Anthelmintics

Of all the drugs found in aquatic systems, antibiotics are present in large quantities given their intensive use in human and veterinary medicine and aquaculture [110,111]. Being that they are in surface/waste waters in dissolved form, their environmental effects have received considerable attention over the years [52,54,112]. Among the commonly used antibiotics are tetracyclines, which are broad-spectrum drugs effective against gram-positive and gram-negative bacteria [55,56]. Their ecological effects on various non-target aquatic organisms following acute exposure to high concentrations have been most extensively studied [63,64,66]. However, Almeida et al. [68] demonstrated that exposing adult zebrafish to four sub-lethal concentrations of oxytetracycline hydrochloride (0, 0.1, 10, 10,000 µg/L, environmental concentration in Table 1 [113]) for two months (see Table 1) increased total protein levels; reduced lipid levels, causing a rise in consumed energy; and decreased the activity of total glutathione, glutathione S-transferase, and catalase, highlighting important oxidative damage at the whole-body level. The biochemical responses observed were concentration-dependent and showed that long-term exposure to oxytetracycline at sub-lethal concentrations can cause a stress condition that results in: the increase in protein synthesis, a reduction in the levels of utilized energy, and changes in the levels of antioxidant enzymes that depend on the oxidative-stress intensity. Total protein and lipid levels have been confirmed as valuable biomarkers for understanding the health of zebrafish, since they are the main constituents of the animal’s body and their quantities typically change in stressful conditions [114,115]. Nevertheless, long periods of exposure can cause a reduction in the activity of antioxidant defences (glutathione and catalase) [68]. This induces oxidative damage, probably due to the prolonged exposure to the drug and its resulting accumulation in the tissues, leading to a reduction of the enzymatic activity [69]. Indeed, it is known that the response of antioxidant enzymes depends on the intensity of the oxidative pressure, and that an overload of the antioxidant defence system can occur in conditions of oxidative stress. The particular results of this study suggest that there may be changes in the antioxidant capacity of the organism due to an incremental rise in the number of reactive oxygen species (ROS) that can cause molecular, cellular and tissue damage [116,117,118,119]. Moreover, glutathione is one of the main components of *Danio rerio’s* extensive antioxidant system and, whether acting independently or in cooperation with glutathione peroxidase, that makes this fish a vertebrate model of choice in studies evaluating redox biology [119].

**Figure 1:**
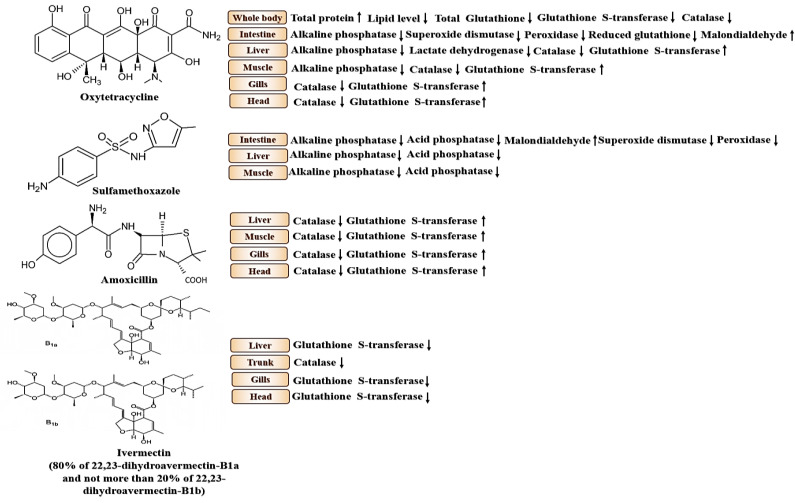
Schematic representation of the effects of antibiotic drugs on the biochemical responses of *D. rerio* adults. The arrow pointing up indicates “up-regulation” while the arrow pointing down indicates “down-regulation”.

Other authors have previously compared the effects of oxytetracycline and sulfamethoxazole (at concentrations of 420 ng/L and 260 ng/L, respectively, for six weeks and environmental concentration in Table 1 [120]) on hydrolytic (alkaline and acid phosphatase) and antioxidant enzymes (superoxide dismutase, peroxidase, reduced glutathione) in intestinal, liver and muscle samples [69]. Significant changes were only identified in the alkaline- and acid-phosphatase activity: the former reduced with both drug treatments, while for the latter this was only the case with sulfamethoxazole. In another study, the same authors fed adult zebrafishes again with sulfamethoxazole and oxytetracycline, but at therapeutic concentrations for six weeks (100 and 80 mg/kg body weight per day) [121]. The results showed that the activities of the digestive enzymes in the intestine were higher for both antibiotic treatments. Lower alkaline and acid phosphatase activities were found in fishes treated with oxytetracycline, highlighting that long-term use of antibiotics caused adverse systemic effects on fish gut health. As key biomarkers, these enzymes are typically involved in the response to diverse types of stress in a variety of aquatic organisms [3,4,122,123]. The changes identified in the study by Zhou et al. [69] support the view that they are possibly important biological indicators, even in the face of the type of stress reported. Moreover, these enzymes are vital components of the non-specific immune system and, in this case, are indicators of an impaired immune function and a confirmed increased inflammatory state. In terms of the antioxidant enzymes, there was a decreasing trend in the intestinal tissue with both pharmacological treatments, but there were no changes in the muscle and liver samples. This highlights that the intestine is probably the more sensitive organ, as it is the first one to be affected. This is confirmed by: (1) the fact that the antibiotics induced the down-regulation of the number of the gut’s goblet cells, which are endowed with defensive functions, (2) the up-regulation of the transcriptional levels of the inflammatory cytokines TNF-α and IL-1, and (3) the drastic changes in the composition of the physiologic bacteria. This study plays an important role because it analyses the long-term biological effects of drugs at ng/L concentrations, highlighting the ecological risks of both oxytetracycline and sulfamethoxazole. However, at different concentrations, it may be that the intestine is not the affected organ. If this is the case, even though this study confirmed that outcomes can depend on the exposure and the tissue analysed, reaching a conclusion of this type requires further experimentation that tests a variety of drug concentrations and analyses biochemical responses in different tissues. This is especially the case if we consider that other authors testing the sulfamethoxazole at different concentrations (50, 100 and 500 mg/L) and over shorter exposure times (3 and 14 days) have not found significant effects on the antioxidant enzymes’ activity in the whole-body of adult zebrafish [124] (Table 1). In this context, an important study was performed by Oliveira et al. [66], who compared the effects of oxytetracycline and amoxicillin (environmental concentration in Table 1 [125]), which is another antibiotic (penicillin-like AB) used extensively in human medicine and aquaculture to treat infections [126]. Comparing the effects of these drugs at the same concentrations and in a wider variety of tissue types than in previous studies, the authors confirmed a general reduction in the amount of antioxidant enzymes present in their specimens. In particular, they tested the sub-lethal effects of the two drugs on the enzymatic responses of catalase, glutathione S-transferase and lactate dehydrogenase in head, muscle, liver and gill samples following short-term exposure (96 h) at 0.1, 10, 25, 50 and 100 mg/L concentrations (Table 1). They found that both antibiotics reduced the levels of catalase and increased those of glutathione S-transferase. With respect to the former, the response was greater in the liver than in the other tissues, because the enzyme is more elevated physiologically due to the fatty acid catabolism-dependent up-regulation of hydrogen peroxide. Meanwhile, glutathione S-transferase activity was lower in the muscle, where it is limited physiologically due to the negligible detoxifying function of this tissue. The response of the lactate dehydrogenase enzyme was down-regulated only by oxytetracycline in the liver (see Table 1). To improve the knowledge of the effects of anthelmintics drugs on enzymatic reactions, the same authors subsequently analysed acetylcholinesterase, glutathione S-transferase and lactate dehydrogenase responses after 96 h of exposure to another drug recently used also to treat COVID-19 disease, ivermectin [127,128]. They exposed adult fish at concentrations of 10, 20, 40, 60, 80, 100 and 200 μg/L [105] (data and environmental concentration showed in Table 1 [129,130]) by examining various biochemical responses in different tissues. In particular, acetylcholinesterase was evaluated in the head, lactate dehydrogenase in the muscle, and glutathione S-transferase in the liver and gills. They identified no changes in acetylcholinesterase in the head samples, a meaningful inhibition of glutathione S-transferase in the gills and liver, and no changes in lactate dehydrogenase in the muscle (see Table 1). One possible explanation for the down-regulation of the glutathione S-transferase activity concerns the observed oxidative stress triggered by the drug, which can deplete the amount of intracellular glutathione and, therefore, render it less available for conjugation with the enzyme. A similar study was performed by [106], who analysed the chronic effects of ivermectin (10, 20, 40, 60, 80, 100 µg/L) after 96 h by testing catalase, glutathione S-transferase and acetylcholinesterase activity in head and trunk tissues (Table 1). Their results confirmed a general reduction in the antioxidant response following the treatment, but the changes were only significant for the catalase and glutathione S-transferase activity. In detail, catalase was inhibited in the trunk at a 25 µg/L concentration, but this was only the case for glutathione S-transferase in the head at the highest concentrations tested, confirming the negative impact on the antioxidant system (see Table 1). Thus, it seems clear that these pollutants influence the oxidative response, even after a short exposure time. In conclusion, although the data reported in the literature regarding the effects of antibiotics and anthelmintics on biochemical responses have been obtained from different biological matrices at different concentrations and times of exposure, it is clear that antioxidant enzymes (also fairly common to most chemical challenges) are valuable bioindicators in understanding the adverse effects of these drugs. Figure 1 summarizes the biochemical responses studied to date of the antibiotics on the different tissues of *D. rerio* adults.

**Table 1:** Biochemical responses in zebrafish exposed to antibiotic and anthelmintics drugs.

Drug	Environmental Concentrations	Concentration/ Time Exposure	Samples	Biomarker Analysed	Biochemical Responses	Reference
Oxytetracycline	ng/L to µg/L [113]	0, 0.1, 10, 10,000 µg/L for Two Months	Whole Body	Total Protein; Lipid; Total Glutathione; Glutathione S-Transferase; Catalase; Acetylcholinesterase; Lactate Dehydrogenase	(+) Total Protein (−) Lipid Level, Total Glutathione, Glutathione S-Transferase, Catalase	[68]
Sulfamethoxazole Oxytetracycline	259,6 ng/L and 350 ng/L Respectively [120]	260 ng/L 420 ng/L for Six Weeks	Intestine, Liver, Muscle	Superoxide Dismutase; Peroxidase; Reduced Glutathione; Alkaline Phosphatase; Acid Phosphatase	(−) Alkaline Phosphatase, Acid Phosphatase, Antioxidant Enzymes	[69]
Sulfamethoxazole Oxytetracycline		100 and 80 mg/kg for Six Weeks	Intestine, Liver, Muscle	Amylase; Lipase; Malondialdehyde; Superoxide Dismutase; Peroxidase; Reduced Glutathione; Alkaline Phosphatase; Acid Phosphatase	(+) Malondialdehyde (−) Peroxidase, Superoxide Dismutase, Reduced Glutathione, Alkaline Phosphatase, Acid Phosphatase	[121]
Sulfamethoxazole		50, 100 and 500 mg/L for 3 and 14 Days	Whole-Body	Glutathione Peroxidase; Glutathione Reductase; Glutathione S-Transferase; Lipid Peroxidation	No Significant Effects	[124]
Amoxicillin Oxytetracycline	6 and 340 ng/L, Respectively [125]	0,1, 10, 25, 50, 100 mg/L for 96 h	Head, Muscle, Liver, Gills	Catalase; Glutathione S-Transferases; Lactate Dehydrogenase	(−) Catalase, Glutathione S-Transferase, Lactate Dehydrogenase	[66]
Ivermectin	25 up to 60 ng/L [129,130]	10, 20, 40, 60, 80, 100, 200 μg/L for 96 h	Head, Muscle, Liver, Gills	Acetylcholinesterase; Glutathione S-Transferases; Lactate Dehydrogenase	No Change in Acetylcholinesterase, Lactate Dehydrogenase (−) Glutathione S-Transferase	[105]
Ivermectin		10, 20, 40, 60, 80, 100 µg/L for 96 h	Head, Trunk	Catalase; Glutathione S-Transferase; Acetylcholinesterase	(−) Catalase Activity, Glutathione S-Transferase No Change in Acetylcholinesterase	[106]

(−) decrease; (+) increase.

### 3.2. Antiepileptics and Antipsychotics

**Figure 2:**
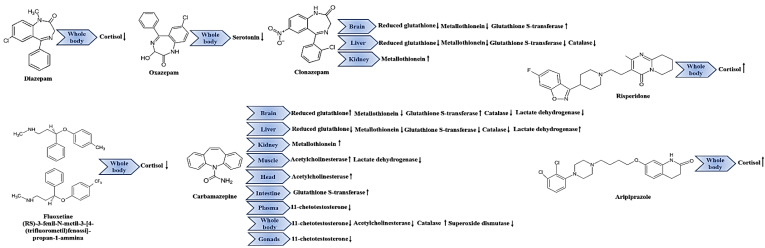
Schematic representation of the effects of antiepileptic and antipsychotic drugs on the biochemical responses of *D. rerio* adults. The arrow pointing up indicates “up-regulation” while the arrow pointing down indicates “down-regulation”.

The World Health Organization (WHO) recently estimated that more than 300 million people today suffer from a mental disorder (WHO, 2018). Over the years, the use of drugs for the treatment of anxiety, psychotic syndromes, mood disorders and depression has increased, leading to higher levels in the aquatic systems. Among different biomarkers, cortisol, a glucocorticoid hormone, is an important parameter for checking the health status of animals, because it plays a relevant role in the different biological processes linked to stress, including the immune response, the osmoregulation, the metabolism and reproduction [131,132]. Zebrafish also use cortisol as a primary stress response hormone [133], and so several authors have evaluated its levels after subjecting the animal to the relevant drugs. De Abreu et al. [134] exposed adult zebrafish to diazepam (0.88 μg/L, 16 μg/L and 160 μg/L) and fluoxetine (1 μg/L, 25 μg/L and 50 μg/L) for 0, 15, 60 and 240 min (environmental concentration in Table 2 [36,41,42,135]). They then analysed the effects on whole-body homogenates (Table 2), demonstrating that only diazepam at intermediate concentrations (16 μg/L) and fluoxetine at environmental concentrations (1 μg/L) have significant effects on cortisol levels and, therefore, on the stress axis function (Table 2). A subsequent study by the same authors showed that even 15 min of exposure to a fluoxetine concentration of 1µg/L reduced cortisol levels in the specimens, confirming a blocking effect on the neuroendocrine stress axis of zebrafish [136]. One of the mechanisms by which cortisol interferes with osmoregulation processes is in the stimulation of the gill Na^+^/K^+^ ATPase [137]. By measuring ion flux levels in fish, the authors demonstrated that the drug reduced the influx of Na^+^ and K^+^ and, therefore, the cortisol levels blocking the osmoregulatory effects triggered by stress. Short-term fluoxetine exposure can thus have effects on the central nervous system. These findings have been confirmed by other authors, who compared the effects of fluoxetine with those of diazepam, assessing the biochemical responses of the fish following exposures to 50 μg/L and 16 μg/L of both drugs for 15 days [138]. It was demonstrated that a long period of exposure reduced cortisol levels in all cases (Table 2), probably blocking the cortisol response to acute stress [134,136]. It is important to note that the observed effects on cortisol levels may also be correlated with behavioural changes, as observed in an innovative study performed by Egan et al. [139], who analysed the effects of fluoxetine (100 μg/L) on the behavioral and biochemical responses of zebrafish. Their work demonstrated an anxiolytic response linked with a decrease in cortisol levels in the whole-body, highlighting an important possible change in the fishes’ states of anxiety (Table 2). Another drug used to treat antiepileptic and antipsychotic disease is oxazepam, whose biochemical effects on zebrafish have been studied by Vossen et al. [140,141] (environmental concentration in Table 2 [142,143]). These authors reported that oxazepam, at different concentrations (7 µg/L and 0.57 µg/L) and for different exposure times (7–28 days and 9 days), reduced the turnover of serotonin in both their male and female specimens by having an impact on the brain neurochemical levels, even without affecting the levels of cortisol (Table 2). Treatment with traditional benzodiazepines (such as oxazepam) causes a dose-dependent decrease in monoamine extracellular neurotransmitters, such as serotonin [144], while monoaminergic signaling is involved in the brain networks activated under conditions of stress and anxiety [145,146]. Stressful situations evoke the release of serotonin in the brain, and benzodiazepines can suppress this peak [147]. In the study by Vossen et al. [141], the absence of changes in cortisol levels in the presence of oxazepam could be due to the type of animal used, i.e., wild or laboratory (see Table 2). Contrary to this finding concerning the effects of oxazepam in whole-body cortisol levels, different results were obtained testing aripiprazole. De Alcantare Barcellos et al. [148], exposed, for the first time, adult zebrafish to five different concentrations of this antipsychotic drug (0.0556, 0.556, 5.556, 55.6 and 556 ng/L) for 15 min (environmental concentration in Table 2 [149]). They demonstrated increases in cortisol levels at all concentrations except two (0.556 and 556 ng/L), highlighting that this drug released into the environment can interfere with the neuroendocrine axis and decrease the stress response of exposed fish (Table 2). In agreement with these authors, further work showed that adult zebrafish exposure to risperidone (0.00034 μg/L, 85 μg/L, 170 μg/L, 340 μg/L and 680 μg/L) after 15 min (environmental concentration in Table 2 [42,150]) can cause increases in whole body cortisol levels, highlighting an interaction among treatment, stress and time of exposure [151]. However, these results must be interpreted with caution, as the mechanism of action of these drugs on the hypothalamus–pituitary–interrenal (HPI) axis of zebrafish is not yet clear, and they refer only to acute exposures. Beyond the drugs discussed so far, the commonly used benzodiazepines, which are, therefore, more present in aquatic environments, are clonazepam and carbamazepine [152,153]. The negative effects of these drugs have been observed in different aquatic species [46,154,155]. To verify this in zebrafish, specimens were exposed to carbamazepine or clonazepam, individually or simultaneously, for 96 h at concentrations of 75 µg/L (showed with environmental concentration in Table 2 [39,40]. The biochemical responses were then analysed in brain, liver and kidney tissues, in particular focusing onto the levels of reduced glutathione, metallothionein, catalase and glutathione S-transferase [156]. All parameters were analysed in the liver and brain samples, while only metallothionein levels were evaluated in the kidney tissue, due to the small amount of material available. The results show that glutathione decreased in the liver and increased in the brain, the effects being more noticeable with a single administration of clonazepam. The levels of metallothionein, active as a free radical scavenger, were down-regulated in the liver by all treatments, with a tendency to recover during concomitant drug administration. In the brain, these levels decreased with both treatments, with more pronounced effects during exposure to clonazepam; in the kidney tissue, there were increases for all treatments. It is probable that metallothionein, in addition to the maintenance of metal homeostasis, also plays a role in the sequestration of free radicals confirming the results of Hauser–Davis et al. [157] that suggested an interruption of essential metal homeostasis, showing that exposing *Danio rerio* to other pollutants induces oxidative stress. For other antioxidant enzymes, increases in glutathione S-transferase levels in brain tissue have been observed during carbamazepine treatment, while reductions in glutathione S-transferase and catalase have been shown in the liver during co-exposure (see Table 2). Accordingly, the levels of antioxidant enzymes increase or decrease depending on the tissue analysed and whether there is a single treatment or a co-exposure to drugs. The changes in reduced glutathione and glutathione S-transferase levels indicate the use of the former one as a substrate for the enzymatic activity of the latter one [158], which can lead to permanent cell damage. Reduced glutathione is a tripeptide and the main cellular soluble antioxidant defence [159] and its depletion can lead to a cascade of events which, ultimately, cause cell death [160]. One of the by-products of this cascade is hydrogen peroxide, a key ROS that is degraded very efficiently by catalase, an enzyme capable of converting millions of hydrogen peroxide molecules into water and oxygen, thus alleviating the effects of oxidative stress [161]. The findings showed that the brain was the more affected organ, with significant effects caused by oxidative stress in both the liver and brain samples. This was the case with both exposure to a single drug and co-treatment, demonstrating that clonazepam and co-administration cause changes in oxidative stress responses [156]. This confirms that drugs of the type analysed in this section are involved in oxidative stress mechanisms in both animal models [162] and humans [163]. They are also responsible for an imbalance between the production of ROS and the antioxidant system, triggering damage to cells and tissues. The effects of carbamazepine exposure have also been tested by Da Silva Santos et al. [164], who exposed animals to 0, 10 and 10,000 μg/L of the drug for 63 days. They then analysed its behavioral, genotoxic, histopathological and biochemical effects on the specimens’ muscles, head, gills, liver and intestine (see Table 2), in particular focusing onto the activity of acetylcholinesterase (muscles and head), catalase (gills and liver), lactate dehydrogenase (gills, liver and muscles) and glutathione S-transferase (intestine, gills and liver) at the biochemical level. The results showed that the activity levels of the different enzymes changed per tissue and exposure, and that the same enzyme could have an opposite effect in different tissues. The level of acetylcholinesterase in the animals’ muscles and head rose after exposure to higher drug concentrations, highlighting the onset of apoptotic events that depended on acetylcholinesterase, the release of free radicals and conditions of oxidative stress [165,166,167]. Catalase levels in the liver and gills decreased, suggesting an inability to cope with the drug-induced level of pro-oxidants. Glutathione S-transferase also increased in the gills, probably due to the fish coming into direct contact with the aqueous-based carbamazepine. The levels of glutathione S-transferase decreased in the intestine, thereby demonstrating a tissue-dependent response. There was a reduction in the amount of lactate dehydrogenase in the gills and muscles, but levels increased in the liver, indicating a change in the anaerobic metabolism and the histological structure of the hepatic and extrahepatic tissue. A very recent study analysed the effects after 45 days of exposure to carbamazepine (1, 10 and 100 μg/L) on the whole body of *Danio rerio* adults studying acetylcholinesterase, catalase, superoxide dismutase and glutathione S-transferase activities (Table 2). The authors highlighted the decreases in acetylcholinesterase, glutathione S-transferase and superoxide dismutase activity at high drug concentrations along with the increases in catalase and glutathione S-transferase at low drug concentrations [168]. The different trends of these enzymatic activities, which have been shown to be concentration dependent, are probably due to a condition of high oxidative stress at high exposure concentrations with a consequential suffering of the organism, unlike the lower concentrations in which the antioxidant system still responds to increase the levels of enzyme released into the circulation. Beyond this, carbamazepine (10 μg/L) also had an inhibiting effect on 11-ketotestosterone levels in the whole body, the plasma and the tests of male fishes after 67 days of drug exposure [169]. The authors suggested that there is an impact on testicular androgen production in zebrafish after chronic exposure, but it seems more probably a general effect in the organism due to stress and cell death, rather than a pure endocrine response. These results confirm those of Galus et al. [170] who, while not observing significant variations in estradiol levels in females, suggested and confirmed a sex-dependent effect of the drug. The biochemical responses described in this paragraph about the effects of antiepileptic and antipsychotic drugs on the biochemical responses of *D. rerio* adults are summarized in Figure 2.

### 3.3. Antidyslipidemics

**Figure 3:**
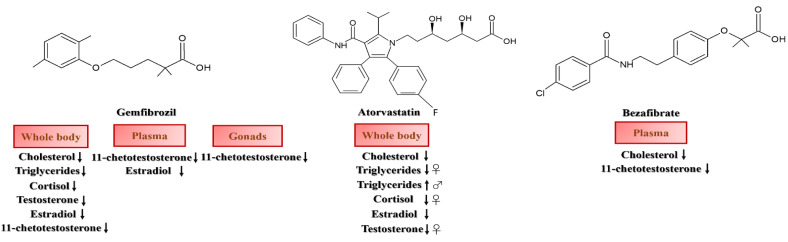
Schematic representation of the effects of antidyslipidemic drugs on the biochemical responses of *D. rerio* adults. The arrow pointing up indicates “up-regulation” while the arrow pointing down indicates “down-regulation”.

Arteriosclerosis and cardiovascular diseases are among the widespread pathologies in human beings today, and they have notable levels of mortality. They are commonly treated with lipid-lowering drugs [171,172] such as gemfibrozil and atorvastatin, which have been widely demonstrated to have a negative impact on aquatic organisms [173,174,175,176]. Over a period of 30 days, Al-Habsi et al. [177] studied the effects of gemfibrozil (16 μg/g) and atorvastatin (0.53 μg/g) on adult zebrafish (environmental concentration in Table 3 [178]), analysing the levels of cholesterol, triglycerides, cortisol, testosterone and estradiol in their whole body (Table 3, Figure 3). Triglyceride levels decreased in the female specimens in all individual drug and co-administrated treatments; they decreased in the males when they were treated with gemfibrozil and rose following exposure to atorvastatin or co-exposure to both drugs. Cortisol levels in the female animals decreased following treatment with atorvastatin or co-treatment with both substances; conversely, there were no changes in the males. Finally, testosterone levels decreased in the females across all treatments, but remained unchanged in the males. Overall, estradiol levels decreased with all treatments in both the females and males. These hormonal changes may be related to alterations in the cholesterol/triglyceride balance, because steroid hormones require cholesterol as a precursor [179,180]. These authors also examined the molecular effects of gemfibrozil and atorvastatin on their fish specimens, demonstrating: (1) the up-regulation of PPARA, coding for the peroxisome proliferator-activated receptor α in the females, but not the males, which is consistent with the greater reduction in the triglyceride levels, (2) the drug-induced up-regulation of PPARG, coding for the Peroxisome proliferator-activated receptor γ, (3) the atorvastatin-triggered up-regulation of SREPB1 (sterol regulatory element-binding protein), coding for a transcriptional activator of fatty acids and the triglyceride synthesis, (4) the drug-induced, male-specific up-regulation of CYP3A6, coding for the cytochrome P450 3A6 protein involved in the detoxification of endogenous substances and xenobiotics and (5) the drug-induced, male-specific up-regulation of Atrogin-1, whose protein product is responsible for resistance to the toxic effect of statins. Fraz et al. [169] and Galus et al. [170] revealed the same findings, confirming the inhibition effects of gemfibrozil on 11-ketotestosterone in their male and female specimens (Table 3, Figure 3). These studies highlighted gender-dependent effects, substantiating a lipid-lowering role played by these drugs in the zebrafish species. They also highlighted a link between the impact on lipid regulation and the production of steroid hormones, identifying possible negative effects on the reproductive capacity of the species and, therefore, its survival.

Literature reports on other drugs utilized to lower cholesterol and triglyceride levels concern the use of fibrates [181,182,183], which are commonly found in aquatic systems due to human usage [36,184]. Velasco-Santamaría et al. [185] exposed male zebrafish to different concentrations of bezafibrate (1.7, 33 and 70 mg/g of food, environmental concentration in Table 3 [186]), analysing cholesterol and 11-ketotestosterone levels after 48 h and 7 and 21 days. The authors demonstrated a time-dependent decrease in cholesterol and 11-ketotestosterone in the specimens’ plasma (Table 3, Figure 3). This highlighted a cholesterol-lowering effect (decrease in plasma cholesterol and 11-KT levels), as well as the ability to interfere with the endocrine system and, therefore, with the reproductive success of the species. Interestingly, molecular analyses of the exposed fish revealed effects at the gene expression level, in particular on: (1) the down-regulation of *PPARA* and *PPARG* after 48 h of treatment and the up-regulation of *StAR*, coding for the steroidogenic acute regulatory protein involved in the transport of cholesterol to the inner mitochondrial membrane and (2) *CYP17A1A*, coding for the cytochrome P450 family 17 subfamily A member 1 protein, which is a monoxygenase involved in the steroidogenic pathway. As a consequence, this study shows that lipid regulators, such as fibrates, are significant and dangerous endocrine disruptors in zebrafish specifically and, probably, in fish in general.

**Table 3:** Biochemical responses in zebrafish exposed to antidyslipidemic drugs.

Drug	Environmental Concentrations	Concentration/ Time Exposure	Samples	Biomarker Analysed	Biochemical Responses	Reference
Gemfibrozil Atorvastatin	1500–2100 ng/L and 15–44 ng/L, Respectively [178]	16 μg/g 0.53 μg/g for 30 Days	Whole Body	Cholesterol; Triglycerides; Cortisol; Testosterone; Estradiol	(−) Cholesterol (−) Triglycerides (−) Cortisol (−) Testosterone (−) in Estradiol	[177]
Gemfibrozil		10 μg/L for 67 Days	Whole Body, Plasma, Gonads	11-Chetotestosterone	(−) 11-Chetotestosterone	[169]
Gemfibrozil		0.5 and 10 μg/L for 6 Weeks	Plasma	11-Ketotestosterone; Estradiol	(−) 11-Ketotestosterone	[170]
Bezafibrate	Up to 3.1 [186]	1.7, 33 and 70 mg/g for 48 h, 7 and 21 Days	Plasma	Cholesterol; 11-Chetotestosterone	(−) Cholesterol 11-Chetotestosterone	[185]

(−) decrease; (+) increase.

### 3.4. Analgesic, Antipyretic and Anti-Inflammatory Drugs

**Figure 4:**
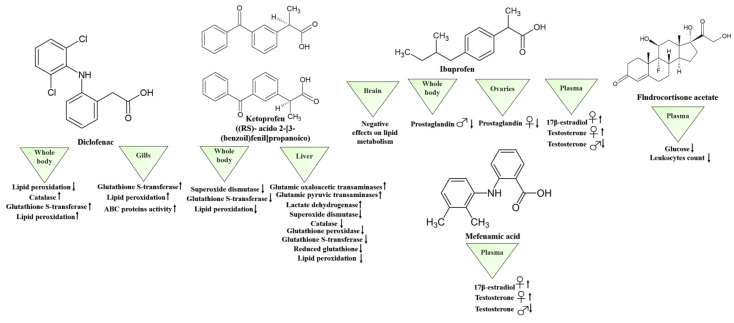
Schematic representation of the effects of analgesic, antipyretic and anti-inflammatory drugs on the biochemical responses of *D. rerio* adults. The arrow pointing up indicates “up-regulation” while the arrow pointing down indicates “down-regulation”.

Among the PhACs identified in surface waters are non-steroidal anti-inflammatory drugs (NSAIDs), which can have dangerous effects on aquatic organisms, even at low concentrations [30,35,58,187,188,189]. A very common NSAID is diclofenac, which is widely used in diseases such as osteoarthritis, rheumatoid arthritis and abdominal cramps [49]. The effects of this drug on different aquatic species are well known [190], but only a few studies have analysed its impact on the biochemical responses of zebrafish. In this regard, important research has been conducted by Praskova et al. [191], who evaluated the effects on adult specimens of 28-day exposures to diclofenac at different sub-chronic concentrations (0.02, 5, 15, 30 and 60 mg/L and environmental concentration showed in Table 4 [191]). Using the whole body as their sample, the authors examined the responses to oxidative stress, in particular the activity of glutathione S-transferase and reduced glutathione and the levels of lipid peroxidation. The latter was the only parameter to show significant decreases following exposure to the drug (Table 4, Figure 4). Lipid peroxidation is a well-known biomarker of the oxidative damage caused to membranes by different types of toxic substances. These substances can lead to cell and tissue damage and, therefore, to developmental malformations and alterations [192,193]. These outcomes may, however, depend on the capacity of diclofenac to inhibit cyclooxygenases (prostaglandin–endoperoxide synthase). Unlike the previous study, de Carvalho Penha et al. [194] performed sublethal tests by subjecting adult zebrafish individuals to concentrations of 3 mg/L and 2 μg/L of diclofenac for 96 h (see Table 4). The biochemical analysis was performed on liver and gills and in particular, glutathione-S-transferase, the percentage of ABC protein activity, lipoperoxidation and ethoxyresorufin-O-deethylase activity were analyzed. Significant increases were observed only in gills concerning glutathione-S-transferase activity, the percentage of ABC protein activity and lipoperoxidation, showing that, perhaps, the gills are the primary and the most affected organs, being in interface between fish and water. However, this conclusion can only be applied to short time drug exposure, a time in which the diclofenac, incorporated by the gills, can rapidly degrade and not reach the liver. Beyond diclofenac, Rangasamy et al. [195] examined the effects on adult zebrafish of ketoprofen (environmental concentration in Table 4 [58]), another NSAID commonly found in aquatic environments [196,197,198]. After exposing their specimens to different drug concentrations (1, 10 and 100 µg/L) for 42 days, the authors evaluated the enzymatic responses in the liver of: glutamic oxaloacetic transaminases, glutamic pyruvic transaminases, lactate dehydrogenase, including superoxide dismutase, catalase, glutathione peroxidase, glutathione S-transferase, reduced glutathione and lipid peroxidation. In detail, exposure to ketoprofen increased the levels of glutamic oxaloacetic transaminases, glutamic pyruvic transaminases and lactate dehydrogenase in a dose- and time-dependent manner; conversely, the levels of antioxidant enzymes and lipid peroxidation decreased at all exposure concentrations, with the maximum achieved after 42 days (Table 4, Figure 4). This study confirms that the liver’s detoxification and biotransformation activities make it the most important metabolizing organ and, therefore, one of the main defences against drug pollution [199]. It also highlights that, in addition to the enzymes involved in oxidative stress [200], the levels of aspartate transaminase and alanine aminotransferase are also important given their involvement in the metabolism of proteins and amino acids in fishes. In zebrafish, the alteration of these enzymes means a stress condition, liver tissue damage and high levels of oxidative stress. As the removal of pharmaceutical products by WWTPs is notably inefficient, it is essential to develop methodologies that will improve the work these plants do; one of the solutions proposed is the use of UV radiation as a disinfectant to reduce the concentration of drugs [201,202,203,204]. However, it has been reported that these photolysis processes can produce chemical compounds that are even more dangerous than the original drugs [51,205], meaning that knowing their effects is vital. For this reason, Diniz et al. [206] analysed the oxidative response (glutathione S-transferase, superoxide dismutase, catalase and lipid peroxidation) in the whole body after exposure to ketoprofen and diclofenac (1 mg/L) and to their photodegradation products under UV irradiation (Table 4). The sampling for each drug was conducted according to the time it took for their photoproducts to form (for diclofenac, after 1.5 and 5 min, and for ketoprofen, after 7.5 and 60 min). The study revealed that diclofenac’s photolysis products were the most toxic, causing increases in the levels of glutathione S-transferase, catalase and lipid peroxidation; ketoprofen, on the other hand, showed lower toxicity levels. This research provides the perspective of an experimental analysis aimed at evaluating a drug in its original structure. However, taking also into account the chemical changes that may cause the drug to have different effects could help to improve the technologies used by WWTPs. Along with diclofenac and ketoprofen, ibuprofen is another of the main drugs detected in aquatic systems [207,208,209,210,211]. Ji et al. [212] studied the effects of five NSAIDs (acetylsalicylic acid, diclofenac, ibuprofen, mefenamic acid and naproxen) at three different concentration (10, 100 or 1000 μg/L and environmental concentration showed in Table 4 [213]) on hormone production in plasma samples after 14 days of exposure by adult zebrafishes. Ibuprofen and mefenamic acids, at 10 and 100 μg/L, showed a significant increment in 17β-estradiol and testosterone concentration in females, while that of testosterone decreased among male fish. These data highlighted that ibuprofen could modulate hormone production in a sex-dependent way, probably causing adverse effects on reproduction and the development of offspring. This result is different from that obtained by Morthorst et al. [214]. In fact, these authors exposed zebrafishes to moderate concentrations of ibuprofen (21, 201 or 506 μg/L) for seven days and observed that prostaglandin E2 (PGE2) levels in whole-body homogenates of males and ovaries of females decreased in a dose-response relationship, while male 11-ketotestosterone and ovarian 17β-estradiol levels remained unchanged. Ibuprofen also did not affect vitellogenin levels (Table 4). This study showed that, although ibuprofen reduces PGE2 levels in male and female zebrafish, it does not appear to have consistent effects on other reproductive parameters studied. Ibuprofen is also another drug for which variable inter- and intra-specific toxicities have been demonstrated [209,215]. Indeed, in humans and other mammals, ibuprofen undergoes extensive chiral inversion from the R to the S form [216], with side-effects reported for the former [217]. To understand the impact on fishes, Zhang et al. [218] performed a relevant and innovative study investigating the effects of the chiral form of ibuprofen (environmental concentration in Table 4 [218]) on the brain tissue of this species. In particular, zebrafishes were exposed to concentrations of 5 μg/L for 28 days, and lipidomic analyses were then performed. Significant changes were observed in the levels of lipids, such as glycerophospholipids, sterol lipids, phenolic lipids, fatty acids, glycolipids and sphingolipids, highlighting the negative effects of ibuprofen on how the brain metabolizes them and, therefore, on the composition of biological membranes, the inflammatory and cardiovascular responses, and the cerebrovascular disease (Table 4, Figure 4). Furthermore, it was found that the toxicity of ibuprofen is stereoselective, which may be important information that enables WWTPs to modify the chemical structure of drugs and, therefore, their reactivity [51,205,219]. The knowledge of all these elements is essential because, if drugs change structure when they reach the aquatic system, it is vital to understand and analyse their effects on biodiversity. Other scientific studies have examined the activity of analgesics, antipyretics and anti-inflammatory drugs in plasma samples after a 21-day exposure to a commonly prescribed corticosteroid, fludrocortisone acetate (between 0.006 and 42 μg/L and environmental concentration in Table 4 [220,221]) [222]. The results of this work revealed increases in glucose levels and decreases in the proportion of different leukocytes (lymphocytopenia, Table 4 and Figure 4) as a consequence of chronic stress, confirming that corticosteroids are responsible for regulating plasma glucose levels, and can also modulate immune responses in fish species [223,224,225]. This research also identified transcriptional changes in the biomarker genes involved in gluconeogenesis, the immune response and the circadian rhythm. In relation to the latter, the authors found transcriptional changes in: *PEPCK1*, coding for the gluconeogenesis-involved phosphoenolpyruvate carboxykinase; *SOCS3*, coding for the STAT-induced STAT inhibitor suppressor of the cytokine signalling 3 protein; *G6PCA*, coding for the glucose-6-phosphatase controlling the homeostasis of glucose blood levels; *PXR*, coding for the steroid- and xenobiotic-sensing nuclear pregnane X receptor; *SLCO2A1*, coding for the prostaglandin-binding solute carrier organic anion transporter family member 2A1 protein; *PER1A*, coding for the photoperiodism-involved period circadian clock 1A protein; *NR1D1*, coding for the circadian rhythm-coordinating nuclear receptor subfamily 1 group D member 1 protein; and *HSD17B3*, coding for the testosterone-producing hydroxysteroid 17-β dehydrogenase 3.

## 4. Conclusions

A review of the various scientific papers concerning the impact of human drug pollution on biochemical responses in the tissues of adult zebrafish has allowed us to integrate a great deal of information which would otherwise remain fragmentary, if considered on an individual basis. Undoubtedly, the most difficult task was to reorganize data obtained from studies which reported different concentrations and exposure times for the same drugs whilst analyzing different tissues. It proved an almost impossible task to compare results obtained by the various authors in scientific literature and to provide useful indications regarding possible minimum levels of drug release in aquatic environments.

**Figure 5:**
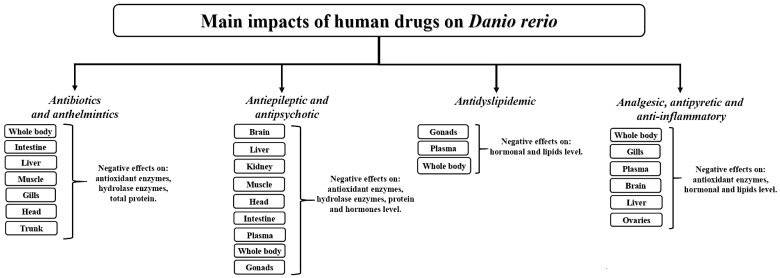
Conceptual diagram illustrating the main impacts of human drugs on *D. rerio*.

The goal of this review was to analyze, for the first time, all the scientific literature on this topic by unifying information from various authors, thus identifying the main impacts of human drugs on D. rerio adults (Figure 5). It is evident that individual scientific studies are not sufficient to provide a reliable indication of limits, in particular as it has emerged that the biochemical effects observed on adult zebrafish individuals depend on various factors, such as tissue analyzed, duration of exposure to the drug, drug concentrations and sex of individual. Moreover, although oxidative stress appears to be a shared causal factor among the drugs examined in this paper, some of the still poorly elucidated biochemical and molecular aspects require further investigation. As an example, clarification is needed on whether drug-triggered impairment of lactate dehydrogenase (a NAD+ regenerating enzyme) ultimately acts through the NADH/NAD+ redox imbalance to determine cellular oxidative stress, resulting in macromolecular oxidative damage (as proposed by Wu et al.) [226]. Another problem which has become evident is that drugs can be found in aquatic environments either in their original or their chiral structure (e.g., enantiomers), further explaining why it is extremely difficult to regulate the use of drugs, with serious social, environmental and economic consequences [227]. The economic growth of a nation depends on it being healthy and disease-free, and our constant interactions with the environment affect the quality of our lives and state of health. The WHO defines the term “environment” (in relation to health) as all the physical, chemical and biological factors external to a person, and all related behaviors (World Health Organization, 2006). There is an urgent need, therefore, for guidelines to regulate the production, sale, use and disposal of drugs. Although guidelines do currently exist, such as those provided by the European Medicines Agency (EMA) (which provides estimates of the ecological risk of drugs based on bioaccumulation potential and production levels in order to limit the release of human drugs for marketing authorization) or the US FDA (which requests results of environmental assessments for all newly developed drugs), there is relatively little information available. Undoubtedly, efforts to reduce the environmental impact of human drugs on aquatic systems needs to be greatly intensified. In conclusion, we would suggest to authors of future studies to test the same concentrations of various drugs for the same time intervals on the same tissues. This would provide comparable and useful information to assist in drafting emission limits for human drugs in aquatic systems and in improving purification systems.

## Figures and Tables

**Table 2:** Biochemical responses in zebrafish exposed to antiepileptic and antipsychotic drugs.

Drug	Environmental Concentrations	Concentration/Time Exposure	Samples	Biomarker Analysed	Biochemical Responses	Reference
Diazepam Fluoxetine	0.04–0.88 µg/L [41,42] and 0.012 to 1 µg/L, respectively [36,42,135]	Diazepam: 88 μg/L, 16 μg/L and 160 μg/L; Fluoxetine: 1 μg/L, 25 μg/L and 50 μg/L for 0, 15, 60, 240 min	Whole Body	Cortisol	(−) Cortisol	[134]
Fluoxetine		1 μg/L for 15 min	Whole Body	Cortisol Ionic Fluxes	(−) Cortisol Alteration Effect on Ionic Fluxes	[136]
Diazepam Fluoxetine		50 μg/L, 16 μg/L for 15 Days	Whole Body	Cortisol	(−) Cortisol	[138]
Fluoxetine		100 μg/L for 2 Weeks	Whole Body	Cortisol	(−) Cortisol	[139]
Oxazepam	ng/L to μg/L [142,143]	7 μg/L for 7 and 28 Days	Whole Body	Cortisol; Serotonin	No Effects on Cortisol level (−) Serotonin turnover	[140]
Oxazepam		0.57 μg/L for 9 Days	Whole Body	Cortisol	No Effects on Cortisol Level	[141]
Aripiprazole	5.56 ng/L [149]	0.0556, 0.556, 5.556, 55.6 and 556 ng/L for 15 min	Whole Body	Cortisol	(+) Cortisol	[148]
Risperidone	0.00034 μg/L [42,150]	0.00034 μg/L, 85 μg/L, 170 μg/L, 340 μg/L and 680 μg/L for 15 min	Whole Body	Cortisol	(+) Cortisol	[151]
Carbamazepine Clonazepam	0.002 to 11.5 μg/ L [40]; 145 ng/L [39]	75 µg/L for 96 h	Brain, Liver, Kidney	Reduced Glutathione; Metallothionein; Catalase; Glutathione S-Transferase;	(−) Glutathione in Liver (+) Glutathione in Brain (−) Metallothionein in Brain and Liver (+) Metallothionein in Kidneys (+) Glutathione S-Transferase in Brain (+) Glutathione S-Transferase in Liver (−) Catalase	[156]
Carbamazepine		0, 10 or 10,000 μg/L for 63 Days	Muscle, Head, Gills, Liver, Intestine	Acetylcholinesterase; Catalase; Glutathione S-Transferase; Lactate Dehydrogenase	(+) Acetylcholinesterase (−) Catalase (+) Glutathione S-Transferase (+) Lactate Dehydrogenase in Liver (−) Lactate Dehydrogenase in Brain and Muscle	[164]
Carbamazepine		1, 10 and 100 μg/L for 45 Days	Whole Body	Superoxide Dismutase; Acetylcholinesterase; Catalase; Glutathione S-Transferase	(−) Acetylcholinesterase, Glutathione S-Transferase, Superoxide Dismutase (+) Catalase, Glutathione S-Transferase	[168]
Carbamazepine		10 μg/L for 67 Days	Whole Body, Plasma, Gonads	11-Chetotestosterone	(−) 11-Chetotestosterone	[169]
Carbamazepine		0.5 and 10 μg/L for 6 Weeks	Plasma	11-Ketotestosterone; Estradiol	(−) 11-Ketotestosterone	[170]

(−) decrease; (+) increase.

**Table 4:** Biochemical responses in zebrafish exposed to analgesic, antipyretic and anti-inflammatory drugs.

Drug	Environmental Concentrations	Concentration/ Time Exposure	Samples	Biomarker Analysed	Biochemical Responses	Reference
Diclofenac	0.02 mg/L [191]	0.02, 5, 15, 30, and 60 mg/L for 28 Days	Whole Body	Glutathione S-Transferase; Reduced Glutathione; Lipid Peroxidation	(−) Lipid Peroxidation	[191]
Diclofenac		3 mg/L and 2 μg/L of for 96 h	Gills, Liver	Glutathione S-Transferase; Percentage Of ABC Proteins Activity; Lipoperoxidation; Ethoxyresorufin O-Deethylase Activity	(+) Glutathione S-Transferase Activity, Percentage Of ABC Proteins Activity, Lipoperoxidation	[194]
Ketoprofen	Up to 1.0 μg/L [58]	1, 10 and 100 µg/L for 42 Days	Liver	Glutamic Oxaloacetic Transaminases; Glutamic Pyruvic Transaminases; Lactate Dehydrogenase; Superoxide Dismutase; Catalase; Glutathione Peroxidase; Glutathione S-Transferase; Reduced Glutathione; Lipid Peroxidation	(+)Glutamic Oxaloacetic, Transaminases, Glutamic Pyruvic Transaminases, Lactate Dehydrogenase; (−) Other Parameters	[195]
Ketoprofen Diclofenac and Their Photodegradation Products		1 mg/L for Ketoprofen, 7.5 and 60 min; for Diclofenac 1.5 and 5 min	Whole Body	Glutathione S-Transferase; Superoxide Dismutase; Catalase; Lipid Peroxidation	(+) Glutathione S-Transferase, Catalase, Lipid Peroxidation; (−) Catalase, Glutathione S-Transferase, Superoxide Dismutase, Lipid Peroxidation	[206]
Acetylsalicylic Acid, Diclofenac, Ibuprofen, Mefenamic Acid and Naproxen	<0.02, 0.15, 0.07, and 0.07 μg/L in Germany Rivers [213] 0.269 μg/L, 0.793 μg/L, 3.528 μg/L, 1.390 μg/L, and 0.326 μg/L in Korea Rivers	10, 100 or 1000 μg/L for 14 Days	Plasma	17β-Estradiol; Testosterone	(+) 17β-Estradiol, Testosterone in Females; (−) Testosterone in Male	[212]
Ibuprofen		21, 201 or 506 μg/L for 7 Days	Whole Body and Ovaries	Prostaglandin; 11-Ketotestosterone; 17β-Estradiol	(−) Prostaglandin E2 (PGE2); No Change 11-Ketotestosterone, 17β-Estradiol	[214]
Ibuprofen	5 μg/L [218]	5 μg/L for 28 Days	Brain	Lipidomic Analysis	Changes Lipid Levels	[218]
Fludrocortisone Acetate	Low ng/L Range in Surface and Ground Waters; Up to Hundreds of ng/L in Influent/Effluent of WWTPs [220,221]	0.006 to 42 μg/L for 21 Days	Plasma	Glucose; Leukocytes Count	(−) Glucose, Leukocytes	[222]

(−) decrease; (+) increase.

## Data Availability

Not applicable.
